# The Illness Experience of Long COVID Patients: A Qualitative Study Based on the Online Q&A Community Zhihu

**DOI:** 10.3390/ijerph19169827

**Published:** 2022-08-09

**Authors:** Yi Wang, Sheng Bao, Yubing Chen

**Affiliations:** School of Journalism and Communication, Huaqiao University, Xiamen 361000, China

**Keywords:** long COVID patients, illness experience, stigma

## Abstract

Long COVID is a public health problem that cannot be ignored, and it is critical to understand the long COVID patients’ living situations and support this group through their illness narratives. This study is based on grounded theory, and coded the self-produced texts of long COVID patients on the largest online Q&A community in China, Zhihu APP, in an attempt to explore the illness experiences of long COVID patients in China and to understand how they adapt to their illness and reconstruct their lives. The results show that patients face not only the threat of pain from the illness itself, but also social stigma and discrimination. Patients turn their illness experiences into motivation to move forward and reconstruct self and life by ‘pushing forward the biographical flows again’, ‘impression management’ and ‘self-compassion’. These findings can help policy-makers and medical institutions to provide timely and appropriate policy support and psychological assistance to patients with long COVID, to create a supportive and inclusive social environment, and to reduce discrimination and stigma against them.

## 1. Introduction

The COVID-19 pandemic has had a huge impact on the health and well-being of people around the world; not only is the pandemic itself (as a highly contagious disease) threatening, but the potential for longer-term effects of COVID-19 infection are also becoming a problem that cannot be ignored. A growing body of new evidence suggests that some people who contract Coronavirus Disease 2019 (COVID-19) do not make a rapid or full recovery—so-called “long COVID” [1,2]. As defined by the World Health Organization (WHO), long COVID occurs in individuals with a history of probable or confirmed COVID-19 infection, usually 3 months from the onset of COVID-19 with symptoms that last for at least 2 months, which cannot be explained by an alternative diagnosis [3]. According to the National Health Commission of the People’s Republic of China, as of 1 April 2022, there were 153,232 cumulative confirmed cases of COVID-19 in China [4], and although there are no definitive data on the incidence of long COVID in China, based on existing studies in other countries approximately 10–30% of patients with COVID-19 develop long-term symptoms [5,6]. Given this, long COVID will affect at least 15,000 people in China, and the numbers are bound to grow further as the pandemic continues. This potential impact represents a significant threat to public health security.

It is evident that long COVID is a condition that requires sustained attention and multi-disciplinary research. At present, most studies on long COVID have focused on the medical and psychological fields, but few studies have focused on the self-narrated illness experiences of long COVID patients, and even fewer have focused on this group in China. Therefore, this paper attempts to examine the illness experiences of long COVID patients in China, and to explore their life situations from the patients’ perspective.

Since Strauss and Glaser’s pioneering work [7], there has been a growing interest among humanities scholars in the study of the meaning of illness and the patients’ experience. Over the decades, research on illness narratives have explored the subjectivity of the illness experience, patients’ coping strategies, and the interaction between the illness experience and social structures, etc. [8,9,10].

With the COVID-19 pandemic, some scholars have shifted the focus of their research to the experiences of people with COVID-19 and those who recovered during the pandemic. Bogusz et al.’s qualitative study of Polish recovered patients with COVID-19 found that the patients were burdened with institutional and emotional struggle during the entire process of being ill, despite a mild course, and the disease institutionalization significantly modifies the illness experience of the recovered patients [11]. In a study by Sahooa et al. of patients hospitalised with COVID-19 in India, they found that patients’ mental health was at great risk, with anxiety, depression, insomnia and internalized stigma being the main issues [12]. Not coincidentally, Santiago et al.’s study of adults who had recovered or were recovering from acute infection found that participants in the study had similar levels of psychological distress, but they could understand and cope with the uncertainty of illness through high levels of health information-seeking behavior [13].

However, there is still a paucity of studies on the illness experience of patients with COVID-19, and even less on long COVID patients. As a totally new disease, there are still many unknowns that await further research. Analyzing patients’ perspectives from their narratives is essential to support this group and understand their current living situations. Therefore, this study attempts to explore patients’ illness experiences through self-produced narratives of illness on China’s largest online question and answer community, Zhihu, and to understand how they have adapted to their illness and rebuilt their lives. It is hoped that this study will add information to the emerging field of ‘long COVID’ and echo the existing research.

## 2. Materials and Methods

### 2.1. Data Source and Collection

Grounded theory is both a methodology and a method used in qualitative research, originating from the social sciences but widely used in education and health research [14]. The focus of grounded theory is building on primary sources from which experience can be generalized and eventually arrive at a theory with general applicability. Grounded theory was chosen for this study because of its ability to develop concepts and models from information ‘grounded’ in the data, serving to explain the content of texts, rather than being based on a priori theory or assumption [15,16]. Grounded theory enabled the construction of a model of long COVID patient’s illness experience in China, and its applicability can be verified in subsequent studies.

Specifically, self-narratives of long COVID patients on the Zhihu App were selected as the data for analysis. As one of the largest online Q&A communities in China, as of 1 April 2022, the topic of “COVID-19 Pandemic” on Zhihu has generated 248,000 discussions. After browsing and screening related questions, this paper selected two questions with answers of relatively good quality: “What are the sequelae of COVID-19?” and “What happens to people who have had COVID-19 now?”, and all answers under the two questions up to 1 April 2022 were obtained via Python, a total of 908. After screening the data and eliminating invalid answers (advertisements, non-patient self-reports, less than 100 words, etc.), the final 64 answers remained. Five answer texts were randomly retained for coding saturation test, and the rest were collated and imported into NVivo 12 as raw data for the coding of grounded theory.

### 2.2. Data Source and Collection—Data Analysis

**Figure 1:**
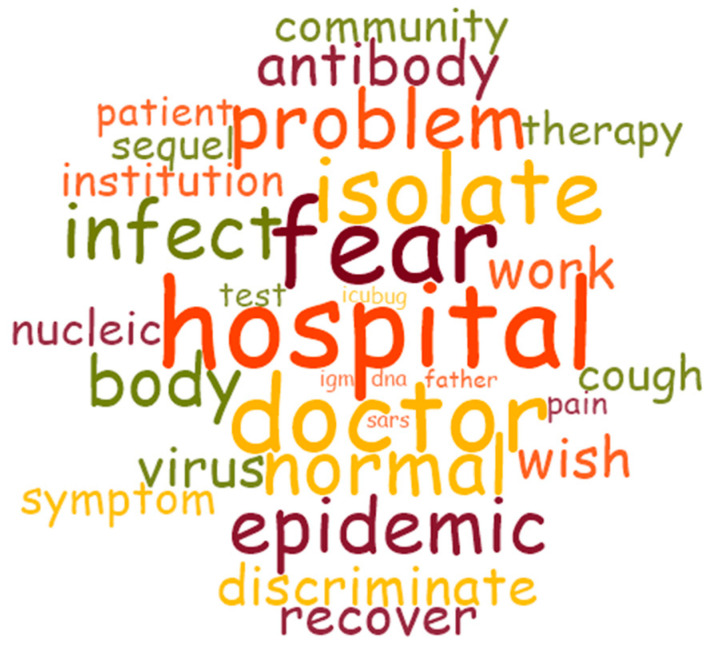
High Frequency Word Cloud.

Grounded theory takes the approach that open coding, axial coding and selective coding operate in parallel to continuously analyze and correct the original texts [17]. Before formal coding, the original texts were imported into NVivo 12 for data pre-processing using the word frequency query function, and the high frequency word cloud map was obtained after eliminating irrelevant words (Figure 1).

#### 2.2.1. Open Coding

Open coding, as a fundamental part of grounded theory, analyses the collected self-reported texts of the COVID-19 patients word by word, conceptualizing and categorizing the sentences or paragraphs that can be used for coding. In the first step, the original texts were tagged and 47 free nodes (a1–a47) were obtained, as shown in Table 1. After comparison and analysis, some of the free nodes were found to have cross-semantics or identical semantics, which were merged and further categorized to obtain 15 initial categories (A1–A15).

#### 2.2.2. Axial Coding and Selective Coding

The coding of initial categories is relatively scattered and the relationships between them are not clear. For this reason, axial coding is operated according to the logical relationships and relevance of the open codes in order to analyze the inner link between initial categories, then regrouping on that basis. With further integration of the 15 initial categories, 6 main categories (B1–B6) were obtained, and on the basis of several analyses and condensation towards open coding and axial coding, 2 core categories (C1–C2) were derived after further generalization and integration.

#### 2.2.3. Coding Saturation Test

The saturation test is the final step of grounded theory. In this paper, the saturation test was conducted on five randomly retained texts. No new concepts or categories were found after the three-level coding, and on this basis the analytical mechanism of this paper is considered saturated. The three-level coding system is shown in Table 2.

## 3. Results

### 3.1. The Disordered Body and Life

#### 3.1.1. The Return of the Sick Role

According to the current Diagnosis & Treatment Protocol for COVID-19 in China, COVID-19 patients who meet the following criteria can be defined as “clinically cured” and be discharged: (1) Body temperature is back to normal for more than three days; (2) Respiratory symptoms obviously improved; (3) Pulmonary imaging shows obvious absorption of inflammation; (4) Nuclei acid tests negative twice consecutively on respiratory tract samples such as sputum and nasopharyngeal swabs (sampling interval being at least 24 h) [18]. As the body gradually perceives the long COVID symptoms, the newly discharged patient may realize that he or she has reverted from ‘‘normal’’ to ‘‘sick’’. In contrast to the patient role construction process of “body abnormality—seeking medical help—acquiring patient role”, long COVID patients are confronted with a chronic condition for which no specific treatment or diagnostic guidelines exist in China.

“Thought I’d be discharged and all would be well…only to find out that I seem to have another disease, one for which there is no cure yet …”.

[K,1]

“Now there are many physical sequelae, like constant headaches, dizziness, eye and orbital pain, bloodshot eyes, small dark spots appear when looking at things, easily getting fatigue, even waking up in the morning, tinnitus with pulse sounds and rumbling in the ears. I have been to the hospital many times and have had many tests done on my chest, lungs, head, nose, heart and so on, probably other tests I can’t remember. I feel helpless and pain that no one can understand and nowhere to talk about!”.

[C,1]

When long COVID patients seek medical care, medical treatment technology reduces the “lived body” into an object body, the patient’s experience of illness is less important compared to medical tests and imaging indications, and conclusive diagnosis is made with the help of sophisticated medical equipment and data. The unverifiable subjective experience renders even modern medicine helpless, exacerbating the anxiety of long COVID patients.

“…the doctor unilaterally felt that my headaches were just caused by stress and also suggested me to see a psychiatrist”.

[A,1]

The re-awakening of illness perception is indicative of the COVID-19 survivors moving from “normal” to “sick”.

#### 3.1.2. A Divergent Body-Self

Patients describe the range of physical sensations associated with long COVID symptoms as a different self-perception than before. Most patients express a sense of lack of control over their bodies and that their bodies are “strange and unfamiliar”.

“…My body no longer seems to be mine… My hands sometimes shake uncontrollably as if someone is manipulating my body behind my back…”.

[T,2]

Some long COVID patients stress that they had never thought about how they connected with their bodies before the illness, as in the case of patient J, “*It all seemed to go with the flow*”, but as the long COVID symptoms arrive, patients become increasingly aware that there is a gap between their bodies and their selves, and that their bodies do not necessarily change in response to their sense of self.

“I asked myself over and over again, is this me? Is this really me? I used to run 1 km in the school sports day with ease, but now I can’t even walk up the stairs without panting”.

[Z,2]

The sharp contrast between the existing physical condition and the past physical condition also triggers a negative self-perception of the patients.

“I feel like I have become an 80-year-old granny, feeling useless and powerless ……”.

[W,2]

#### 3.1.3. Stigma and Self-Stigma

Phelan sees stigmatization as exploitation, domination and disease avoidance [19]. Due to the public’s fear of Coronavirus, long COVID patients are seen as potentially “infectious virus carriers” even after they have been treated and discharged from hospital. This stigmatized image is applied to the field of everyday life, creating a sense of fear among other members of society in that field, causing healthy individuals to impose sanctions of social isolation and social exclusion on people with long COVID, and instantly breaking down the system of social relationships that the patients have built and maintained for decades.

“When I meet colleagues, some just say hello at a far distance and walk away, some haven’t spoken to me again so far, some take the mask out of their pockets as soon as they see me and put it on hastily ……”.

[B,3]

In labor and in social interactions, long COVID patients are transformed from “value providers” to “exceeding the bounds” and are rejected by their original group.

“The colleagues who were luckily recovered were basically discriminated against in the unit building, well, in a non-obvious way. And I was reassigned straight to the front line and stopped going to the building”.

[J,3]

This is not an isolated case, as 19 long COVID patients in the collected texts have been invisibly “segregated” in the workplace, and some of them experience occupational discrimination and are implicitly labelled as “inferior labor”.

“I’ve been waiting for months, but still haven’t been hired, and I’ve had my nucleic acid test done so many times that I can’t remember. All the departments are afraid of taking the blame, so they are passing the buck to each other, which I know. They are afraid that I will retest positive, but I have never retested positive and now I am being labelled a ‘plague’ instead”.

[U,3]

Although some patients were previously found to have retested positive in China during regular follow-up strategies for discharged COVID-19 patients, after excluding the possibility of reinfection, it is speculated that the positive signal of viral RNA might be from the “dead” viruses. Current studies point out that discharged COVID-19 patients who retested positive for viral RNA have low or no infectivity [20,21]. However, people with long COVID are still seen as a “potential threat” by the population due to their weak health knowledge.

This discrimination and misunderstanding occurs not only at work, but also among the patients’ friends and families.

“In the six months since I was discharged from hospital, I have had little contact with relatives I used to be close to, let alone visit them”.

[B,3]

“…… My best friend of more than 10 long years said when I was in hospital that she wanted to meet me for a good chat after I was discharged, but when I contacted her, she immediately changed the subject and eventually didn’t meet me ……”.

[V,3]

In addition, in response to the COVID-19 epidemic, China has developed a three-in-one system of individual, community and environmental protection to prevent the spread of the epidemic. The community is the “last mile” of epidemic prevention and control, but the shortcomings of the system and the complexity of grassroots prevention and control have been exposed in the epidemic [22]. Studies have found occasional misinterpretations of epidemic prevention policies in the community, resulting in excessive control and mandatory quarantines [23], while stigma is reinforced by misinterpreting the policies, reinforcing the public’s inference of guilt against long COVID patients.

“My personal and family information is transparent in the community and my unit, and everyone is focusing on me and on guard against me, with frequent nucleic acid tests to prevent my retest positive.”

[R,3]

Public stigma can be extremely damaging to long COVID patients, as they develop self-discrimination and self-stigma after perceiving negative stereotypes, prejudices and discrimination from others.

“…… Now I go out less and less, fearing that I will cause problems and burdens to others ……”.

[I,3]

“……I always felt that I had caused trouble for the country and society, that I had disgraced my parents… When I couldn’t sleep at night, I would blame myself for going out that day, if I hadn’t gone out, I wouldn’t have been infected“.

[M,3]

The self-stigma makes long COVID patients lose their self-worth and see themselves as a ‘‘nuisance’’ and a ‘‘burden’’, forgetting that they are also innocent patients and that no one has the right to discriminate or stigmatize them.

### 3.2. Reconstructing Self and Life

#### 3.2.1. Pushing Forward the Biographical Flows Again

Biographical disruption positions the onset of chronic illness as a major life disruption in which changes to body, self and resources occur [24]. In the case of long COVID sufferers, the illness has brought about a huge change in their lives, forcing them to interrupt their life plans and change their interests. However, it also inspires their sense of exploration and the setting of entirely new social goals.

“I used to love bungee jumping, but now I’ve been advised by my doctor not to try it again due to health reasons … As a result I’m now hooked on embroidery and find I’m quite talented, so I’d like to try out for the city’s embroidery competition next!”

[R,4]

Although the disease brings about a huge change in the lives of people with long COVID, it is also a constant reminder to patients to reorganize the sequence of values in life, with the old desire for money and promotion giving way to the individual’s need to enjoy the present moment and pursue health.

“Even though life is hard now, I have to face the future positively! Being healthy is always the most important thing! To try all the things that I was afraid to try before!”

[H,4]

“In the past, I always thought that I should work more overtime in the company to show myself in front of the leaders and get a promotion and a pay rise as soon as possible. Now I’ve come to realize that there is nothing more important than health, and it’s better to spend more time with myself and my families than to waste time on overtime”.

[W,4]

In this sense, what appears to be a disruption of the life course of the patients is actually a reinforcement of it. The patients readjust to the changes that the illness brings to their lives, from ‘loss’ to ‘gain’, and push forward the biographical flows again [25].

#### 3.2.2. Impression Management

Our identities, however, are developed through an interaction between how we see ourselves and how others conceive of us. Long COVID gives the patients the “sick role”, and it is imperative for them to regain their “healthy” social image and live as normal people. For those who are not yet aware of their condition among those around them, “hiding” has become the norm.

“No one around me at the new school knows that I had COVID-19. I’m the same as everyone else, and my medical check-up at the beginning of the school year was fine, and I don’t say a word about it”.

[M,5]

“If you want to still pretend to be normal, then the most effortless and efficient way is to hide it. Either you hide your condition or hide yourself”.

[T,5]

By “hiding” their illness history, patients with long COVID are able to socialize with others like ordinary people without worrying that they will be shunned out of fear. However, for those already known by their acquaintances to have had COVID-19, “hiding” is no longer effective, and “denial” has become the main theme of their impression management. By denying their poor conditions, they can build a healthy body image and break down the concerns of others about their physical condition.

“When people ask me if I have any after-effects of COVID-19, I choose to deny it ……”.

[N,5]

“When I go to the company gym to work out, some of my colleagues will ask me intentionally or not, “Are you all right? Can you work out?”. I’m not happy inside, but I still smile and say “I’m fine” and then run 5 km to prove myself”.

[B,5]

In addition to denying that their state is not as bad as others think, patients also try to reinforce other aspects of their social identity in order to escape the single “sick role”.

“… I often say when I talk to people that I have a high retirement salary, a filial son, and a healthy grandson who is fatter and taller than children of his age”.

[O,5]

“I saw someone ask me in a private message on Zhihu app ‘How can I make people forget that I had COVID-19′ … I would like to say that although the fact that you had COVID-19 will not change, people’s attitude towards you will change. Now I volunteer in the community on weekends and many people in the community know me and have forgotten that I was once infected with COVID-19, they just say, ‘You’ve worked hard, you’re doing a good job’”.

[Z,5]

Social identity theory asserts that people ascribe different identities to convey who they are and to communicate with the groups to which they belong in certain situations [26].

Long COVID patients are motivated to use multiple social identities, implement positive impression management, and gradually reinforce these in their respective groups, thereby gaining group support and recognition. In turn, group support reinforces the individual’s self-worth and self-identity. In the interaction between the individual and the group, the long COVID patients gradually move away from the single patient identity and rebuild a new social image.

#### 3.2.3. Self-Compassion

Self-compassion is derived from Asian philosophy, and refers to the ability to remain kind and understanding of oneself in the face of negative external events and perceive one’s experiences as part of the experience of a larger humanity rather than seeing them as separating and isolating [27]. For long COVID patients who have undergone a major life change, it is inevitable that their lives and bodies will be disrupted by the disease and that they will briefly wallow in negative emotions. However, individual resilience and mental strength push patients to face everything in their lives with more optimism, and self-compassion is an important way for them to rebuild themselves.

“It may have been hard to adjust at first… but in retrospect, it was no big deal. So many of our public officials and medical staff are at work to confront the epidemic, and my hard work is nothing compared to theirs, so I’m just taking it as a training”.

[G,6]

Self-compassion is not self-pity or being self-centered, nor is it a compromise with the reality of one’s circumstances, but rather an acknowledgement and acceptance of one’s situation. The self-compassion of long COVID patients in a sense not only reaches the state of “acceptance” towards life, but also go on to a state of “transcendence”.

“Now what I already had the ill, instead of weeping and feeling sad every day, I would rather spend my time on more important things and be a more valuable person… I signed up to go to a remote mountainous area to teach in the summer, and when I saw that the children still love learning despite the hardship, I instantly felt that what I was doing was such a meaningful thing”.

[Q,6]

It is true that having long COVID is an “unfortunate” event for them, but it is “fortunate” that the patients’ self-compassion becomes a powerful motivation for them to face up to life and rebuild it. As one patient on the Zhihu App added six months after posting her first answer:

“In the past six months, I’ve been through a lot of dramatic things, I got engaged, then got COVID-19, the groom broke off the engagement, lost my job, got re-employed… Life is always like this, everything doesn’t go smoothly, the most important thing is that your heart is always facing the light, shining bright like the sun!”

[Y,6]

## 4. Discussion

The global pandemic of COVID-19 has had a huge impact on the health and well-being of people around the world and has given rise to many medical and non-medical issues. The “state of exception” under the epidemic is a brutal representation of the fragility and instability of people’s bodies. In this context, it is more relevant to look again at the individual’s life situation and living state, and to explore how life in an abnormal situation can ascend from “zoē” (life common to all living things) to “bios” (life as an individual with quality) [28]. This paper therefore focuses on the experience of illness in the self-written narratives of long COVID patients, in an attempt to examine the life situations behind their narratives and how patients adapt to their illnesses and rebuild their lives.

**Figure 2:**
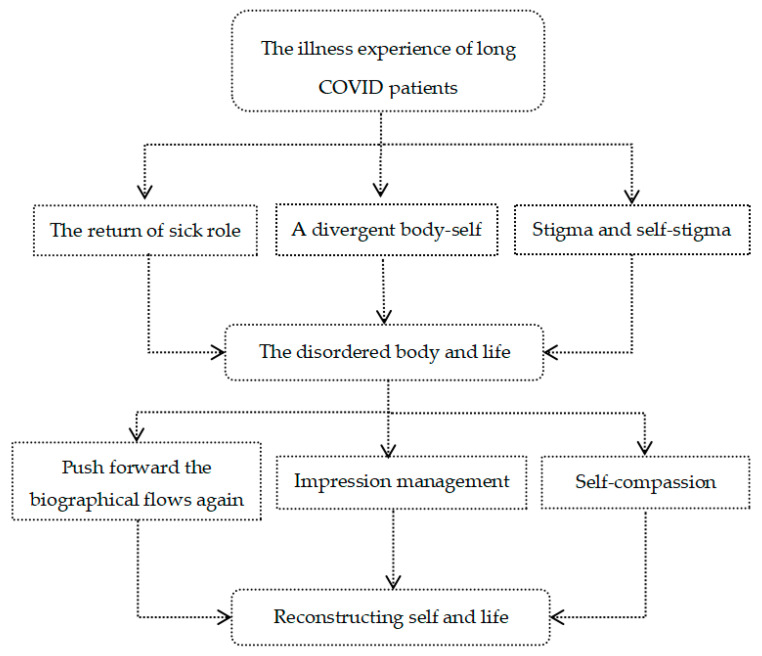
Text Analysis Framework.

Figure 2 is the analysis framework of this study. Our study found that illness made patients take up the “sick role”, and when patients perceived visible or non-visible discrimination at work and social isolation, it exacerbated their internal negative emotions and social avoidance. This is similar to previous quantitative studies, where perceived discrimination was positively associated with negative emotions such as depression and stress [29,30]. For example, in the Section 3.1.3, the public lacks health knowledge, fearing that long COVID patients will retest positive and become infectious again, which leads to suspicion and distance from the patients in social interactions. There is sufficient evidence in the current study to confirm that a positive retest of the discharged COVID-19 patients is clinically non-significant, and there is no evidence to prove that they are infectious [31,32]. Although the Chinese Ministry of Human Resources and Social Security has now issued a circular on the prohibition of employment discrimination against people who have recovered from COVID-19 [33], according to previous studies discrimination persists at the societal level despite relevant laws enacted by the Chinese government to prohibit discrimination against people with infectious diseases such as hepatitis B virus (HBV) and human immunodeficiency virus (HIV) [34,35]. Furthermore, the uncontrolled spread of (mis)information, news and propaganda related to COVID 19 on social media has turned the COVID-19 pandemic into an “infodemic”, further increasing public panic and discrimination against people with COVID-19 [36,37]. How to better communicate health information to the public and stop the spread of misinformation and disinformation about COVID-19 is a crucial issue that needs to be addressed [38].

This qualitative study also found a link between public stigma and self-stigma. Previous research has found that public stigma regarding long COVID patients changes the way they view themselves, which leads to self-stigma, and in turn triggers low self-esteem and low self-efficacy [39,40]. Meanwhile, we found that self-stigma leads to avoidance-based coping strategies, such as in the Section 3.2.2, where long COVID patients tend to use “hiding” or “denial” strategies in an attempt to get rid of the patient’s social image and to tell people around them that they are in fact healthy. While these coping strategies which have been shown in other studies of patients with chronic diseases are shown to be related with a higher quality of life and happiness [41], there is also evidence that patients may avoid seeking medical assistance and social support due to fear of discrimination, which in turn reduces quality of life [42]. Therefore, psychological interventions for long COVID patients are necessary, such as large-scale COVID-19-related psychoeducation programs, while anonymous online counseling may be a good approach given the privacy and accessibility of patients [43,44].

Illness is not the disappearance of life order, but rather the emergence of a new order of life [45]. Although there is no way to completely eliminate the pain experienced by patients, this study found encouraging evidence that patients transform their illness experiences into motivation to move forward and rebuild their lives, and this change in mindset may be an idea for personalized therapies for patients with long COVID. In particular, we found that “self-compassion” played an important role in reducing negative emotions and improving the life quality of patients. This is similar to previous studies, where self-compassion, as an intrapersonal resource, may be an effective means for individuals to cope with tough situations during the pandemic [46,47]. Therefore, future psychological interventions for long COVID patients could try to improve patients’ level of self-compassion and carry out individualized self-compassion training, etc., to help them cope with their plight and maintain their personal emotional health during the pandemic.

Certainly, there are some research limitations in this study, such as collecting the data of patients from only one online community, which may have some impact on the results. Future research could examine the illness experiences of long COVID patients on the social media with more diverse user composition. A mixed-method approach to data collection, such as in-depth interviews with long COVID patients who fit the theme of the study, could also be considered to ensure the rigor of the data. Follow-up studies could also cross-sectionally compare the similarities and differences in patients’ illness experiences across different countries and cultures to explore the underlying social and cultural factors.

## 5. Conclusions

Based on grounded theory, this study analysed and coded the self-produced texts of illness experiences of long COVID patients on the online Q&A community Zhihu through the qualitative analysis software NVivo 12. The results show that patients with long COVID suffer not only from the pain of the disease, but also stigma and discrimination from society. In the meantime, patients turn the illness experience into motivation to move forward and rebuild themselves and their lives through impression management and self-compassion as strategies. This study may help policy-makers to understand the living conditions of long COVID patients in China and develop relevant intervention policies to provide medical support and psychological guidance for them. It is even more important to create a supportive and inclusive social environment, stop the spread of unsubstantiated COVID-19 rumors, convey correct health information, and reduce stigma and prejudice against long COVID patients. After all, as Sontag says, “We are not being invaded. The body is not a battlefield. The ill are neither unavoidable casualties nor the enemy. We—medicine, society—are not authorized to fight back by any means whatever [48]”.

## Figures and Tables

**Table 1:** Open Coding (Excerpt).

Original Texts (Excerpt)	Free Node (Excerpt)
Now there are many physical sequelae, like constant headaches, dizziness, eye and orbital pain (a1), bloodshot eyes, small dark spots appear when looking at things, easily getting fatigue (a2), even waking up in the morning, tinnitus with pulse sounds and rumbling in the ears. I have been to the hospital many times and have had many tests done on my chest, lungs, head, nose, heart and so on (a3), probably other tests I can’t remember. I feel helpless and pain that no one can understand and nowhere to talk about (a4)! Feeling like a completely different person from my old self (a5) and needing to readjust to my current body in order to continue living (a6). When I meet colleagues, some just say hello at a far distance and walk away, some haven’t spoken to me again so far, some take the mask out of their pockets as soon as they see me and put it on hastily …… (a7)	a1 Ongoing pain
	a2 Body weakness
	a3 Invalid medical tests
	a4 Pain inside
	a5 Split between past and present
	a6 Trying to adapt to the body
	a7 “Social death”

**Table 2:** The Three-level Coding System.

Open Coding	Axial Coding	Selective Coding
Initial Category	Main Category	Core Category
A1 Physical abnormalities of subjective perception	B1 The return of sick role	C1 The disordered body and life
A2 Medical signs of objective examination		
A3 Sense of lossing control of the body	B2 A divergent body-self	
A4 The unfamiliar body		
A5 Employment discrimination	B3 Stigma and self-stigma	
A6 Social isolation		
A7 Perceptions of being discriminated		
A8 Internalised stigma		
A9 Set new goals	B4 push forward the biographical flows again	C2 Reconstructing self and life
A10 Change the old values		
A11 Conceal the illness	B5 Impression management	
A12 Deny the sequelae		
A13 Rebuild the social image		
A14 Be kind to self	B6 Self-compassion	
A15 Accept the imperfect self		

## Data Availability

The data generated during and/or analyzed during the current study are available from the corresponding author on reasonable request.
